# Feasibility and acceptability of a structured counselling approach to address lifestyle-related risk factors during pregnancy and postpartum care: an exploratory longitudinal pilot feasibility study

**DOI:** 10.1186/s40814-026-01955-y

**Published:** 2026-11-10

**Authors:** Manuela Bombana, Katharina Kraufmann, Katja Krug, Michel Wensing

**Affiliations:** 1 Department of General Practice and Health Services Research, University Hospital Heidelberg, Im Neuenheimer Feld 130.3, Heidelberg, 69120 Germany; 2 Department for Prevention, AOK Baden-Württemberg, Presselstraße 19, Stuttgart, 70191 Germany

**Keywords:** Feasibility, Pilot study, Pregnancy, Lifestyle-related risk factors, Counselling, Implementation

## Abstract

**Background:**

Lifestyle-related risk factors (LRRFs) are major contributors to adverse maternal and child outcomes. Despite clear international guidelines, counselling on these topics is inconsistently implemented in routine care. Structured tools may support comprehensive, consistent counselling, yet evidence for multi-domain approaches in maternal health is limited.

**Methods:**

This exploratory longitudinal pilot feasibility study evaluated a structured counselling approach to support structured communication on five LRRFs (smoking, alcohol, nutrition, physical activity, and stress) in German outpatient midwifery and gynecological practice. Quantitative provider and session-level data were complemented by open-ended provider feedback collected at the end of the field phase. Ten providers participated (eight midwives, two gynecologists). Feasibility outcomes comprised provider recruitment and retention, the number and planned duration of documented counselling sessions, fidelity of delivery, and completeness of documentation. Provider acceptability was assessed using structured questionnaire items addressing perceived usefulness, usability and flexibility, complemented by open-ended feedback on barriers and facilitators. Quantitative data were analyzed descriptively; open-ended responses were reviewed manually and grouped into descriptive categories.

**Results:**

The ten providers documented 256 counselling sessions. On average, providers addressed approximately two LRRF domains per session, most commonly nutrition, physical activity, and stress. High fidelity, defined as addressing ≥ 4 domains per session, remained below the predefined 75% threshold at all three measurement points (MPs) (coverage of four or more domains at MP1: 14.9%; MP2: 11.1%; MP3: 7.4%), suggesting selective rather than comprehensive application. Documentation completeness was high. Acceptability ratings were positive: clarity of materials (mean 6.9/10), efficiency (6.8/10), and perceived usefulness (5.8–6.9/10) were rated moderately high. Usability and flexibility were rated as moderate, reflecting time constraints and the need to adapt the structure to client needs. Qualitative feedback highlighted strengths in topic organization and communication flow, alongside barriers related to limited time and contextual fit.

**Conclusions:**

Within this small, purposively recruited sample, the structured counselling approach could be applied during routine counselling encounters and provider acceptability was generally positive, although comprehensive coverage of LRRF domains was uncommon. These findings are specific to the present pilot setting and support further refinement and evaluation in larger and more diverse healthcare settings, including direct assessment of patient perspectives.

**Trial registration:**

Not applicable.

**Supplementary Information:**

The online version contains supplementary material available at https://doi.org/10.1186/s40814-026-01955-y.

## Introduction

Lifestyle-related risk factors (LRRFs) such as smoking, alcohol consumption, poor diet, physical inactivity, and psychosocial stress are globally recognized contributors to maternal and child morbidity including intrauterine growth restriction, preterm birth, fetal alcohol spectrum disorders, gestational diabetes, hypertensive complications, and impaired child development and mortality [1–6].

The global prevalence of LRRFs during pregnancy remains high. Around 4.2–16.6% of women worldwide smoke during pregnancy, with rates up to 18.9% in some European countries [7–9]. In Germany, about 14% of pregnant women report alcohol use [10]. Maternal overweight and obesity at conception affects worldwide 43.8% [11], while micronutrient deficiencies remain common in disadvantaged groups [12]. Psychosocial stress is increasingly recognized as a determinant of adverse outcomes [13]. However, routine assessment and support remain inconsistently implemented across health systems. While recent national surveys in high-income settings report that the majority of women are asked about mental health during antenatal care, substantial gaps and disparities persist—particularly outside such contexts [14–17]. Equity considerations reinforce this rationale. LRRFs disproportionately affect women with lower education, income, or health literacy, as well as migrant and minority groups [18–20]. Without attention to equity, interventions risk widening disparities. Structured approaches, if inclusively designed, may help standardize care delivery.

International guidelines, including those from the World Health Organization (WHO), the American College of Obstetricians and Gynecologists (ACOG), and the National Institute for Health and Care Excellence (NICE), emphasize counselling on smoking cessation, alcohol avoidance, nutrition, physical activity, and mental health [21–23]. Yet translation into practice remains inconsistent. Providers frequently mention time constraints, discomfort discussing sensitive issues, and lack of structured approaches as barriers [24]. Without user-friendly resources, counselling is often unstructured and variable [24].

Evidence from other fields demonstrates that structured counselling interventions improve fidelity and provider confidence [25, 26]. Diabetes education and smoking cessation protocols are examples where standardized approaches enhance outcomes [27, 28]. In maternal health, however, structured approaches addressing multiple LRRFs are scarce, and evidence on feasibility and acceptability is limited [29, 30]. Digital health approaches illustrate further demand for structured content, yet many apps lack theoretical grounding and integration into practice [31, 32]. A structured counselling approach could be adapted for digital delivery, improving reach and scalability [33].

Various theories and frameworks provide potential explanations for low feasibility of tools. This study was primarily informed by Cognitive Load Theory, which provides a theoretical rationale for structuring information in ways that may reduce unnecessary cognitive demands [34].

Given the high prevalence of LRRFs, gaps in counselling practice, and limited availability of structured interventions, we piloted a modular “book chapter” counselling approach, introduced to providers to support consistent and comprehensive counselling on these domains. In contrast to many existing antenatal lifestyle interventions, which tend to focus on a single behavioral domain such as gestational weight gain [35, 36] or smoking cessation [29, 37], the present approach integrates multiple risk factors. This feasibility study evaluated the feasibility and provider acceptability of applying the approach during routine counselling encounters. By examining both feasibility and acceptability, the study provides essential groundwork for refining the intervention and informing the design of future large-scale evaluations.

## Methods

### Study design and setting

We conducted an exploratory pilot feasibility study with a longitudinal design and three measurement points. Quantitative questionnaire and session-documentation data were analyzed descriptively. Open-ended responses from the final questionnaire were grouped into categories describing perceived strengths and weaknesses of the approach, barriers and facilitators to implementation, difficulties during study participation, and positive aspects of participation. Quantitative and qualitative findings were integrated descriptively in the interpretation of feasibility and acceptability. Reporting was guided by the STROBE Statement for observational studies.

The study focused on feasibility (recruitment, retention, characteristics of counselling sessions, fidelity, completeness of data) and acceptability for providers (clarity, usability, efficiency, perceived usefulness, flexibility and adaptability). The field phase covered a total of three months. Within this period, providers completed three structured measurement points (MP 1–3), interspersed with an active documentation week (MP 2) and a planned three-week break, before continuing with a follow-up measurement (MP 3) to explore whether observed changes persisted at follow-up.

The counselling content itself was not co-created with patients specifically for this study. The study questionnaires were reviewed in two expert consultations. Subsequently, an experienced midwife piloted all phases of the study over one week, including the questionnaires and the book-chapter structure, and her feedback informed minor revisions to the training material before the field phase.

### Intervention

The intervention was the ‘book-chapter structure’, a communication method identified through literature searches in PubMed and Cochrane, supplemented by snowball searching. The method was informed by principles of Cognitive Load Theory and provides a structured approach to communicating selected counselling topics related to smoking, alcohol, nutrition, physical activity, and stress in German midwifery and gynecological practice. Its core elements are to name the topic at the beginning of the conversation, divide the information into clearly signposted subchapters, focus on key information, provide opportunities for questions, and summarize or use teach-back to support understanding. This format is intended to reduce cognitive load for providers, support systematic and consistent delivery of key messages, and align with implementation frameworks emphasizing clarity, standardization, and adaptability. At the same time, the individual chapters can be flexibly combined to accommodate individual needs and client-driven discussions [25, 38].

Patients were not involved in co-creation of the method in this pilot study. Providers received written training material on the method after completion of the baseline measurement. They were then asked to apply the structure in subsequent counselling encounters concerning lifestyle-related risk factors whenever applicable.

### Participants and recruitment

Ten healthcare providers were purposively recruited to maximize variation in profession and years of experience. Specifically, we aimed to include both physicians (obstetricians/gynecologists) and midwives, as well as providers working in primary and secondary care. We also sought variation in experience (from < 5 to > 20 years in practice) to capture diverse perspectives. Inclusion criteria were: (1) active involvement in pregnancy or postpartum care; (2) willingness to review the training materials and apply the approach; and (3) ability to document sessions. Recruitment was initially attempted through a local professional association for midwives, but this approach yielded no responses. The study coordinator subsequently approached midwives and gynecologists through existing professional contacts. Ten healthcare providers were recruited through this approach; because recruitment was based on direct professional contacts, a population-based response rate cannot be calculated. Ten healthcare providers were approached and all ten agreed to participate in the study.

The study participants were healthcare providers. Women receiving counselling were not enrolled as study participants. Providers documented characteristics of the counselling encounters, but no patient-level sociodemographic, clinical or identifiable data were collected. The women receiving counselling were women in all stages of pregnancy and postnatal care at participating sites. No patient data were collected at any point.

## Study procedures and data collection

### Expert consultation

Prior to application, an expert consultation was undertaken to evaluate the study questionnaires with respect to comprehensibility, practicality, and face validity. Based on previous studies, the research team had access to an existing pool of healthcare professionals from gynecology and midwifery, from which two experts were purposively selected based on their professional backgrounds and their relevance to the purpose of the consultation. One was an experienced physician working in outpatient care; the other was an experienced midwife with a Master’s-level health-sciences qualification working in teaching and research. The experts reviewed the questionnaires with respect to comprehensibility, practicality and usability in clinical routine, and face validity. The consultations were conducted separately on 19 and 20 October 2022 and documented using written protocols.

### Training and implementation

At study initiation, providers received a binder containing all study questionnaires and written instructions. The training materials on the book-chapter structure were placed in a sealed envelope together with a link to an instructional video. Providers were instructed to open the envelope only after completion of the baseline measurement (MP 1). After reviewing the training materials, providers were asked to apply the book-chapter structure in subsequent counselling encounters concerning lifestyle-related risk factors whenever applicable.

During the field phase, the study team maintained monthly contact with participating healthcare providers to support study implementation and address study-related questions. No predefined interview guide or data extraction sheet was used for these contacts, and information arising from them was not systematically collected or analysed as a separate data source.

### Session documentation

The field phase took place between November 2022 and January 2023. Providers documented counselling encounters at three predefined measurement points (MP 1–3) using a structured questionnaire (hereafter referred to as the *logbook*). At each measurement point, up to 20 counselling encounters could be documented. The logbook captured, among other aspects, the lifestyle-related risk-factor domains addressed during counselling and whether women asked questions.

MP 1 was the baseline measurement before providers received access to the training materials on the book-chapter structure. MP 2 followed introduction of the book-chapter structure and comprised one week of active documentation, during which providers were asked to apply the structure whenever applicable. A planned three-week break without study documentation followed MP 2, after which MP 3 was conducted as the follow-up measurement to explore whether changes observed after introduction of the method persisted over time.

### Provider-reported feasibility and acceptability

Acceptability and perceived usefulness of the book-chapter structure were assessed after provision of the method and again at the end of the field phase using study-specific provider questionnaires. Closed-ended rating-scale items assessed aspects including clarity and usability of the supporting material, efficiency of information delivery, perceived usefulness, and flexibility and adaptability of the book-chapter structure.

Qualitative information was obtained through open-ended questions in the final provider questionnaire addressing providers’ experiences with the book-chapter structure and the study procedures, including perceived barriers and facilitators to implementation. The following open-ended questions were included: D5, “How do you assess the implementability of the book-chapter structure for counselling on lifestyle-related risk factors in your practice?”; D8, “Did you perceive disadvantages related to the book-chapter structure in counselling on lifestyle-related risk factors? If yes, which?”; D11, “In addition to the aspects mentioned above, were there aspects that you found difficult in connection with the study?”; D12, “In addition to the aspects mentioned above, were there aspects that you found helpful in connection with the study?”; and D13, “Are there any further aspects not yet mentioned that you would like to tell us?”.

The open-ended responses complemented the quantitative feasibility and acceptability measures by providing additional insight into providers’ experiences with the book-chapter structure and the study procedures. Responses were reviewed manually and grouped into categories based on their content. No qualitative analysis software was used.

### Outcomes

Drawing on Bowen’s feasibility framework [39], the feasibility assessment comprised:

1. Provider recruitment and retention
2. Number, planned duration, and characteristics of documented counselling sessions
3. fidelity of delivery, defined as coverage of at least four of the five lifestyle-related risk factor domains, with ≥ 75% of sessions meeting this criterion defined as the prespecified feasibility threshold
4. Completeness of documentation

Recruitment, retention, counselling session characteristics, and completeness of documentation were assessed descriptively, as no formal numerical progression criteria had been prespecified for these outcomes. The prespecified fidelity threshold was retained and was not modified retrospectively after inspection of the data.

Acceptability outcomes were assessed across five domains, drawing conceptually on the Theoretical Framework of Acceptability [40] and on established usability and feasibility constructs [41–43]. These domains included:

1. Clarity of supporting material – whether the provided instructional materials were read and perceived as helpful for understanding and applying the approach (0–10 scale: 0 = not at all helpful, 10 = extremely helpful).
2. Usability – feasibility and practicability of integrating the approach into routine counselling encounters (4-point Likert scale: easy, between easy and neutral, neutral, difficult).
3. Efficiency of information delivery – the extent to which the approach supported clear, concise, and streamlined communication of information (0–10 scale: 0 = not at all helpful, 10 = extremely helpful).
4. Perceived usefulness – the degree to which the modular “book chapter” structure was experienced as helpful for structuring counselling content and ensuring client comprehension (0–10 scale: 0 = not at all helpful, 10 = extremely helpful).
5. Flexibility and adaptability – the extent to which the structured format could be tailored to client needs, including whether counselling techniques shifted during sessions and how strongly the counselling remained focused on the approach itself (0–10 scale: 0 = extremely inflexible, 10 = extremely flexible).

### Data analysis

Quantitative data from provider logbooks and questionnaires were analyzed descriptively using IBM SPSS Statistics version 30. Continuous variables such as session duration were summarized as means, standard deviations and ranges, while categorical variables (e.g. covered topics and fidelity) were reported as percentages and frequencies. Fidelity was defined as coverage of at least four of the five lifestyle-related risk factor domains within a counselling session. For each measurement point, the proportion of sessions meeting this criterion was calculated. A priori, ≥ 75% of sessions meeting the fidelity criterion was defined as the feasibility threshold. Open-ended responses from the final provider questionnaire were reviewed manually and grouped into categories based on their content. These categories reflected providers’ experiences with the book-chapter structure and the study procedures, including perceived strengths and weaknesses, barriers and facilitators to implementation, and difficult and helpful aspects of study participation. Selected provider statements were used to illustrate the category-based findings and were translated from German into English for reporting. No dedicated qualitative data-analysis software was used.

## Results

### Provider recruitment and retention

Ten healthcare providers were recruited. An initial recruitment attempt through a local professional association for midwives yielded no responses; the final sample was recruited through existing professional contacts of the study coordinator.

All ten providers completed the final questionnaire; however, two providers did not complete MP3. Thus, retention for the final provider assessment was complete, whereas longitudinal session documentation was incomplete at the third MP.

As shown in Table 1, providers included midwives (*n* = 8) and gynecologists (*n* = 2). Years of professional experience ranged from 4 to 35, reflecting heterogeneity in expertise.

**Table 1:** Characteristics of participating providers

Provider ID	Profession	Years of experience	Working hours/week
1	Midwife	15	30
2	Midwife	8	45
3	Midwife	4	20
4	Midwife	35	35
5	Midwife	11	30
6	Midwife	21	10
7	Midwife	31	40
8	Gynecologist	24	40
9	Gynecologist	20	12
10	Midwife	21	25

### Characteristics of counselling sessions

The final sample comprised eight female midwives and two female gynecologists. Over the three-month implementation period, a total of 256 counselling sessions were documented across the ten providers (MP1: *n* = 107, MP2: *n* = 81, MP3: *n* = 68). The number of sessions per provider across all MPs ranged from 6 to 40. Planned time for counselling sessions varied in length from 2 to 30 min, with an average duration of 15.7 min. Planned counselling time was longer among midwives than among gynecologists (mean 18.1 vs 6.0 min); because only two gynecologists participated, these descriptive differences were not analyzed inferentially. Details are shown in Additional file 1.

### Fidelity

Table 2 illustrates the covered topics at different measurement points: On average, providers addressed M = 1.82 (SD = 1.28, range 0–5) LRRFs per session. The mean number of topics covered was 1.89 at MP1 (SD = 1.39, range 0–5), 1.69 at MP2 (SD = 1.35, range 0–5), and 1.85 at MP3 (SD = 1.04, range 0–4). At MP1, 45.8% of sessions included 0–1 topics, and 54.2% covered two or more. At MP2, coverage patterns were similar, with a slight increase in sessions covering two topics (28.4%). At MP3, nearly half of all sessions (44.1%) included two topics, while sessions addressing three or more domains declined compared to MP1. Overall, this pattern suggests stable coverage of LRRF domains across time, with a modest shift toward addressing two domains per session at follow-up.

**Table 2:** Covered topics at different measurement points

Number of topics	Covered at MP* 1 (%), *n* = 107	Covered at MP 2 (%), *n* = 81	Covered at MP 3 (%), *n* = 68
0	17.8 (*n* = 19)	19.8 (*n* = 16)	10.3 (*n* = 7)
1	28.0 (*n* = 30)	29.6 (*n* = 24)	23.5 (*n* = 16)
2	19.6 (*n* = 21)	28.4 (*n* = 23)	44.1 (*n* = 30)
3	19.6 (*n* = 21)	11.1 (*n* = 9)	14.7 (*n* = 10)
4	12.1 (*n* = 13)	6.2 (*n* = 5)	7.4 (*n* = 5)
5	2.8 (*n* = 3)	4.9 (*n* = 4)	0

*MP measurement point

Fidelity, defined as coverage of at least four out of five lifestyle domains, was below the predefined threshold of 75% at all measurement points (MP1: 14.9%; MP2: 11.1%; MP3: 7.4%). Instead, most counselling sessions focused on one to two domains, reflecting a more selective rather than comprehensive use of the approach.

Most sessions addressed multiple lifestyle domains, reflecting the interconnected nature of risk factors in maternal health Table 3. Nutrition, physical activity, and stress were the most frequently covered chapters, appearing at MP3 in 57.4% (*n* = 39), 52.9% (*n* = 36) and 41.2% (*n* = 28) of sessions, respectively. Smoking (14.7%, *n* = 10) and alcohol (19.1%, *n* = 13) were addressed less frequently.

**Table 3:** Fidelity of topic coverage across sessions at different measurement points

Topic	Covered at MP* 1 (%), *n* = 107	Covered at MP 2 (%), *n* = 81	Covered at MP 3 (%), *n* = 68
Smoking	18.7 (*n* = 20)	18.5 (*n* = 15)	14.7 (*n* = 10)
Alcohol	20.6 (*n* = 22)	22.2 (*n* = 18)	19.1 (*n* = 13)
Nutrition	51.4 (*n* = 55)	51.9 (*n* = 42)	57.4 (*n* = 39)
Physical activity	47.7 (*n* = 51)	40.7 (*n* = 33)	52.9 (*n* = 36)
Stress	50.5 (*n* = 54)	35.8 (*n* = 29)	41.2 (*n* = 28)

*MP measurement point

### Data completeness

Documentation quality was high, with logbooks completed for a total of 256 sessions. Nine of the ten providers documented all counselling sessions on LRRFs conducted during the study period, while one provider pre-selected specific sessions for documentation.

### Acceptability outcomes

#### Clarity of supporting material

Of the ten providers, eight reported reading the instructional material in full and two reported partial use. On a 0–10 scale assessing helpfulness (10 = extremely helpful, 0 = not at all helpful), the mean rating was 6.9 (SD = 1.3; range 5–9), indicating that the material was generally perceived as useful for understanding and applying the approach.

#### Usability in routine practice

On the Likert scale, one provider rated implementation as *easy*, one as *between easy and neutral*, six as *neutral*, and two as *difficult*. Open-ended responses highlighted both strengths and challenges. Providers valued the structuring function of the approach. For example, one provider stated that “several topics can be separated more clearly” (LE010), while another reported that “the conversations can be better structured” (LE013). In contrast, time demands and the dynamic nature of counselling encounters were described as barriers. One provider noted that the approach “takes a lot of time” (LE019), while another found it difficult to implement “when women introduce many of their own topics in a rather ‘unstructured’ way” (LE010). Another provider described announcing the topic in advance as difficult because “many conversations arise spontaneously from the situation” (LE015). Overall, these findings indicate that while the approach was considered implementable by some providers, ease of use varied and its integration into routine counselling appeared to depend on sufficient time and flexibility during consultations. 

#### Efficiency of information delivery

Assessing whether the approach supported efficient communication, the mean rating was 6.8 (SD = 1.6; range 4.4–9). This indicates that most of ten providers perceived the book-chapter structure as moderately to highly useful for conveying information in a clear and structured manner, although individual ratings varied.

#### Perceived usefulness

Ten providers rated several aspects of perceived usefulness on 0–10 scales. The book-chapter structure was considered moderately helpful for expanding providers’ communication methods (M = 6.2, SD = 1.6, range 3.7–8.6) and for structuring information delivery (M = 6.9, SD = 1.3, range 6.9–8.3). Ratings were somewhat lower for supporting structured counselling overall (M = 6.3, SD = 1.2, range 4.9–9), for making counselling sessions more pleasant (M = 5.8, SD = 0.8, range 4.7–7), and for being generally useful in practice (M = 6.1, SD = 1.0, range 4.4–8). Taken together, these results suggest that providers regarded the approach as particularly valuable for structuring content and enhancing methodological repertoire, while its perceived contribution to session atmosphere and overall usefulness was more modest.

#### Flexibility and adaptability

Providers rated the integration of the book-chapter structure as moderately flexible (M = 5.4, SD = 1.3, range 3.4–8.3). With respect to counselling dynamics, providers indicated that they occasionally shifted between different counselling techniques during sessions (M = 5.1, SD = 2.1, range 2.6–8.7; 0 = at every session, 10 = never). Attention devoted to the counselling technique itself was rated as moderate (M = 5.0, SD = 1.9, range 2.7–9.4). These findings suggest that while the tool could generally be integrated into routine consultations, its use required a degree of flexibility, with providers balancing structured guidance and adaptive responses to client needs.

#### Barriers and facilitators

Further open-ended responses provided additional insight into barriers and facilitators of implementation and providers’ experiences with study participation. Difficulties appeared to depend partly on the counselling context. One provider working mainly with women in the postpartum period stated that “it is difficult to conduct structured conversations” in this setting (LE013), while another reported that implementation was more difficult during home visits because conversations often developed in a less structured manner (LE012). Sufficient consultation time and recurring counselling content were described as facilitating implementation. Participation also prompted reflection on existing counselling practices. One provider reported being reminded “to address or discuss alcohol and cigarette consumption more frequently” (LE010), while another described the opportunity “to reflect on and optimize my counselling structure” (LE014).

## Discussion

### Summary of key results

This pilot feasibility study examined the feasibility and provider acceptability of applying a structured counselling approach addressing LRRFs in pregnancy and postpartum care. All ten providers completed the final provider assessment, although two did not complete session documentation at MP3. Across the three measurement points, 256 counselling sessions were documented. Documentation completeness was high, reflecting strong adherence to study procedures. On average, providers addressed approximately two LRRF domains per session, with nutrition, physical activity, and stress most frequently discussed, while smoking and alcohol were covered less often. Fidelity, defined as coverage of ≥ 4 domains per session, remained below the predefined 75% feasibility threshold, suggesting selective rather than comprehensive use of the book-chapter structure.

Acceptability ratings indicated overall positive perceptions. The instructional material was considered clear and helpful. Usability was rated as moderate, with time constraints and client-driven discussions occasionally limiting structured application. Efficiency and perceived usefulness scored moderately high, particularly for structuring and clarifying counselling content. Flexibility and adaptability were rated as moderate, highlighting providers’ need to balance structured guidance with individual tailoring. Open-ended responses corroborated these findings, emphasizing improved organization and topic separation as key strengths, alongside challenges related to time, consistency, and contextual fit.

Within this small and purposively recruited sample, the findings suggest that the structured approach could be incorporated into counselling encounters, although variation in fidelity and usability, profession- and setting-specific consultation conditions, and provider feedback indicate that further refinement and evaluation across more diverse healthcare settings are required.

### Discussion of key results in relation to existing evidence

Our findings align with evidence from structured interventions in other domains of preventive health [44]. Structured protocols for diabetes self-management and smoking cessation have consistently been associated with improved fidelity, provider confidence, and patient outcomes [35, 45–47]. Evidence from other clinical fields suggests that structured approaches may support more consistent delivery of counselling content [45, 46]. In the present study, providers particularly valued the structuring function of the book-chapter approach, although the low proportion of sessions meeting the fidelity criterion indicates that comprehensive multi-domain coverage was difficult to achieve. A multi-domain approach may better reflect the interconnected nature of LRRFs in maternal health [37, 48].

However, as in prior implementation studies, challenges related to time constraints and the need for flexibility were evident [48, 49]. Rigid adherence to structured formats can conflict with the dynamic nature of clinical encounters, particularly when clients introduce multiple or unexpected concerns [50]. The low proportion of sessions meeting the predefined fidelity criterion—while most sessions covered one to two lifestyle domains—may reflect the difficulty of comprehensive multi-domain coverage within routine counselling encounters. This finding echoes evidence from primary care and maternal health research emphasizing the necessity of balancing structure with adaptability to maintain both provider engagement and contextual relevance [51, 52].

Cognitive Load Theory provides a theoretical rationale for the structured organization of counselling information [38]. In the present study, providers reported that the approach helped structure conversations and separate topics more clearly. These findings are compatible with the intended structuring function of the approach but should not be interpreted as evidence of reduced cognitive load, which was not directly assessed [53, 54]. From an implementation science perspective, clarity, adaptability, and perceived usefulness are relevant determinants of implementation [55, 56]. In the present study, ratings of supporting materials and information delivery were moderately favorable, whereas usability and flexibility showed greater variation.

Taken together, the findings support further refinement and evaluation of the structured, multi-domain counselling approach and highlight the importance of balancing standardization with adaptability in complex counselling encounters.

### Strengths and limitations

This pilot study has several strengths. The longitudinal design enabled the assessment of feasibility across three predefined measurement points, while quantitative data from provider logbooks and questionnaires were complemented by open-ended provider feedback. This provided insight into both the application of the book-chapter structure during counselling encounters and providers’ experiences with its use. The inclusion of both midwives and gynaecologists allowed the approach to be explored across different professional and counselling contexts. Furthermore, implementation during routine counselling encounters provided insight into practical barriers and facilitators that may be relevant for further refinement and evaluation of the structured counselling approach.

Several limitations must also be acknowledged. First, although the book-chapter structure was applied during routine counselling encounters, the small, regionally confined sample and the structured study procedures mean that the study should not be considered an unrestricted real-world implementation study. The provision of training materials and repeated study measurements may also have influenced provider behaviour and perceived acceptability. Second, all fidelity and acceptability data were self-reported, using questions that were not comprehensively validated, which may have introduced social desirability or reporting bias. The absence of direct observation or independent verification could have led to overestimation of fidelity or acceptability. Third, the short implementation period and regular research team contact may have supported adherence, potentially inflating feasibility and acceptability estimates. Fourth, contextual factors such as time constraints, workflow differences, and the dynamic nature of counselling encounters limited the consistent application of the counselling approach. These findings indicate that feasibility may depend on profession- and setting-specific consultation conditions and should therefore be examined across more diverse healthcare settings. Fifth, acceptability was assessed only from the healthcare provider perspective. The study therefore cannot determine how women experienced the counselling approach, whether the structure supported their comprehension, or whether the counselling was perceived as relevant and responsive to their individual needs. Future studies should directly involve women in intervention refinement and assess patient-reported acceptability, comprehension, and perceived relevance. Cognitive Load Theory provides a theoretical rationale for structured information delivery; however, neither intrinsic nor extraneous cognitive load was directly assessed in providers or patients. Patient comprehension and learning were also not evaluated. The present findings therefore should not be interpreted as evidence that the book-chapter structure reduced cognitive load or improved patient learning.

Sixth, the predominance of midwives (8/10 providers) limits the transferability of the findings to gynecological practice. Profession-specific workflows and consultation time may influence feasibility and perceived time burden; these aspects should be examined in samples with greater representation of gynecologists. Seventh, recruitment through existing professional contacts may have introduced selection bias, as participating providers may have differed from the broader provider population in their interest in lifestyle counselling, willingness to engage with the intervention, or relationship with the research team. Finally, equity dimensions—such as socioeconomic or cultural factors influencing counselling delivery—were not systematically assessed and should be addressed in future research.

Despite these limitations, the study provides preliminary information on the feasibility and provider acceptability of applying a structured, multi-domain counselling approach during routine counselling encounters. The findings can inform refinement of the approach and the design of larger evaluations addressing effectiveness, sustainability, and implementation across different healthcare settings. Future studies should also incorporate independent fidelity assessments, explore long-term uptake, and evaluate the approach’s potential to reduce inequities in maternal health counselling. Given the time constraints in routine pregnancy and postpartum care, expecting providers to cover at least four of the five lifestyle domains in a single consultation may not be optimal. Future refinement could examine whether briefly introducing all relevant domains while prioritizing one or two topics for more in-depth discussion according to individual relevance is more compatible with routine consultation constraints. Such an approach should be prospectively evaluated. This approach reflects principles of Cognitive Load Theory and implementation frameworks emphasizing adaptability, and may enhance both fidelity and practical usability.

## Conclusions

In this small, purposively recruited sample of healthcare providers, the structured counselling approach could be applied during routine counselling encounters and was generally perceived as helpful for structuring information, while implementation was constrained by time, consultation context and the need to respond flexibly to women’s questions and priorities. The findings are specific to this pilot setting and cannot be generalized to broader maternal healthcare practice. Further studies with larger and more diverse provider samples, greater representation of gynecologists, direct patient involvement, and patient-reported outcomes are needed to refine the approach and evaluate its feasibility, acceptability, and potential effects across healthcare settings.

## Supplementary Information

Supplementary Material 1.

## Data Availability

The datasets generated and/or analysed during the current study are not publicly available due to institutional policy, but are available from the corresponding author on reasonable request.

## Abbreviations

ACOG: American College of Obstetricians and Gynecologists
FASD: Fetal Alcohol Spectrum Disorders
LRRF: Lifestyle-related risk factor
MP: Measurement point
NICE: National Institute for Health and Care Excellence
SES: Socioeconomic status
WHO: World Health Organization
