# Supplementary material for: Feasibility and acceptability of a structured counselling approach to address lifestyle-related risk factors during pregnancy and postpartum care: an exploratory longitudinal pilot feasibility study
Source: Pilot Feasibility Stud. 2026 Nov 10;12:158. doi: 10.1186/s40814-026-01955-y (PMC13749803; doi:10.1186/s40814-026-01955-y)
Supplement: Supplementary file 1 [file 40814_2026_1955_MOESM1_ESM.docx]

# Additional file 1. Characteristics of counselling sessions

| Provider ID | Planned Duration (min) | Measurement Point | Number of Sessions |  |
| --- | --- | --- | --- | --- |
| 1 | 15 | 1 | 15 |  |
|  |  | 2 | 9 |  |
|  |  | 3 | 10 |  |
| 2 | 15 | 1 | 6 |  |
|  |  | 2 | 8 |  |
|  |  | 3 | 5 |  |
| 3 | 20 | 1 | 10 |  |
|  |  | 2 | 4 |  |
|  |  | 3 | 0 |  |
| 4 | 15 | 1 | 20 |  |
|  |  | 2 | 11 |  |
|  |  | 3 | 6 |  |
| 5 | 30 | 1 | 13 |  |
|  |  | 2 | 11 |  |
|  |  | 3 | 7 |  |
| 6 | 30 | 1 | 6 |  |
|  |  | 2 | 5 |  |
|  |  | 3 | 3 |  |
| 7 | 10 | 1 | 13 |  |
|  |  | 2 | 13 |  |
|  |  | 3 | 14 |  |
| 8 | 10 | 1 | 10 |  |
|  |  | 2 | 8 |  |
|  |  | 3 | 13 |  |
| 9 | 2 | 1 | 4 |  |
|  |  | 2 | 2 |  |
|  |  | 3 | 0 |  |
| 10 | 10 | 1 | 10 |  |
|  |  | 2 | 10 |  |
|  |  | 3 | 10 |  |
